# Quantification of Outdoor Mobility by Use of Accelerometer-Measured Physical Behaviour

**DOI:** 10.1155/2015/910259

**Published:** 2015-12-07

**Authors:** Kristin Taraldsen, Malcolm H. Granat, Jorunn L. Helbostad

**Affiliations:** ^1^Department of Neuroscience, Faculty of Medicine, Norwegian University of Science and Technology, Postboks 8905, 7491 Trondheim, Norway; ^2^School of Health Sciences, University of Salford, Manchester M6 6PU, UK; ^3^Clinic for Clinical Services, St. Olav Hospital, University Hospital of Trondheim, Postboks 3250 Sluppen, 7006 Trondheim, Norway

## Abstract

Hip fractures in older persons are associated with both low levels of daily physical activity and loss of outdoor mobility. The aim was to investigate if accelerometer-based measures of physical behaviour can be used to determine if people undertake outdoor walking and to provide reference values for physical behaviour outcomes related to outdoor mobility. Older persons (*n* = 245), ≥70 years, one year after hip fracture, participated. Six objective measures of physical behaviour collected by an activity monitor were compared with self-reported outdoor mobility assessed with the Nottingham Extended ADL scale. All measures of time and length in upright periods were significantly lower in participants who reported not walking outdoors (*p* < 0.001). A set of cut-off points for the different physical behaviour variables was generated. Maximum length of upright events discriminated best between groups, with 31 minutes as a threshold to determine if a person is more likely to report that they walk outdoors (sensitivity: 0.805, specificity: 0.704, and AUC: 0.871) or 41 minutes or more to determine if a person is more likely to report outdoor walking on their own (AUC: 0.891). Physical behaviour variables from activity monitoring can provide information about patterns of physical behaviour related to outdoor activity performance.

## 1. Introduction

The ultimate aim for many older people is to preserve mobility and stay independent and particularly maintain the ability to walk outdoors. Mobility limitations are however common and have often serious consequences for daily life. Activities of daily living (ADL) instruments often assess if people feel capable of performing activities, for example, outdoor walking [1]. However, for prevention of mobility decline early detection of changes in amount and patterns of actually performed physical activities during daily life is essential.

Low levels of physical activity are common following hip fracture [2, 3] and affect mobility in the short and long run [4]. Few older people return to their prefracture mobility levels after a hip fracture, and many will not be able to walk independently or go out of the house alone [5]. To regain walking ability after hip fracture sufficient mobilisation and physical activity is needed [6].

Activity monitoring using small, body-worn accelerometers enables continuous recording of physical behaviour and can inform on what people actually do during daily life. Thus, such instruments and methods have become important supplements to measures of health and function. Several variables derived from activity monitoring could potentially provide details also on outdoor mobility.

There is little known about the relationship between the monitored free-living physical behaviour and self-reported outdoor mobility. We hypothesised that measures of time and length of upright periods could be used to determine if people walk outdoors. If contextual information about outdoor versus not outdoor walking could be derived directly from the activity monitoring data, this could contribute to early detection of changes in daily life that could be important for prevention of mobility decline in older people in general and hip fracture patients in particular. The aim of this study was therefore twofold: to investigate if accelerometer-based measures of physical behaviour can be used to determine if people undertake outdoor walking and to determine reference values for measures of physical behaviour related to outdoor mobility in this population.

## 2. Methods

### 2.1. Study Design and Recruitment

This was a cross-sectional study using data from the Trondheim Hip Fracture Trial which included 397 community-dwelling subjects with hip fracture aged 70+ years with the ability to walk 10 meters prior to the fracture. In total 245 participants completed the activity monitoring recordings one year after the hip fracture. The study protocol, the intervention, and the main results have been published previously [3, 7–9]. For this paper all participants with a minimum of three complete continuous days of activity monitoring (range 3–7 days) one year following the hip fracture were included. The Trondheim Hip Fracture Trial was approved by the Regional Committee of Ethics in Medical Research (REK 4.2008.335), the Norwegian Social Service Data Services (NSD19109), and the Norwegian Directorate of Health (08/5814). ClinicalTrials.gov registry number was NCT00667914.

### 2.2. Measures

Prefracture function was assessed retrospectively using the Nottingham Extended Activities of Daily Living scale (NADL, 0–66) [10] and the Barthel Index (BI, 0–20) [11]. Other demographic variables included age, sex, and fracture type (intracapsular or extracapsular). Background information one year following the fracture included NADL, BI, cognitive function by the Mini Mental State Examination (MMSE, 0–30) [12], mobility by the Timed Up-and-Go (TUG) (sec) [13] and the Short Physical Performance Battery (SPPB) (0–12) [14], Gait Speed over 4 meters assessed as part of SPPB (m/s), depression by the geriatric depression scale (GDS) (0–15) [15], Grip Strength using a Jamar Hydraulic Hand Dynamometer (kg), and self-reported Fear of Falling by the 7-item Fall Efficacy Scale International (FES-I) (7–28) [16].

*Physical behaviour* was assessed using thigh-worn, single-axis accelerometer-based activPAL monitors (PAL Technologies Ltd., Glasgow, United Kingdom). The monitor is 7 mm (depth) × 53 mm (length) × 35 mm (width) and weighs 20 grams, sampling at 10 Hz, and the battery capacity allows monitoring for more than 7 days. The inertial sensor produces a signal related to thigh inclination and can thus identify posture (sitting/lying from standing) from the position of the thigh [17]. The activity monitors were attached to the front of the participants' nonaffected lower thigh with waterproof tape and worn continuously during the recording period. From the software output of the activPAL information on all upright (standing plus walking) events can be derived for the entire recording period. For this study individual number of upright events and the duration of each of these upright events were used in the analysis. Classification of number of upright events and duration of upright events has previously been shown to be 100% accurate in hip fracture patients [18]. In this study we derived outcomes over each participant's recording period, which varied from three to seven recording days. The six physical behaviour outcomes of interest were mean upright time per day, mean number of upright events per day, mean and median length of upright events, maximum length of all upright events, and variability in upright event lengths (the interquartile range (IQR) measured in minutes).

*Outdoor mobility* 12 months after the fracture was assessed by use of one of the NADL items, where participants, or their next of kin, were asked if they had been walking outdoors during the past 14 days. The possible responses to this item were as follows: not walking outdoors, walked outdoors with personal assistance, walked outdoors alone with difficulty, or did walk outdoors alone.

From the sample, 81 participants reported that they had not been outdoors, 33 did go outdoors with personal assistance, 36 did go outdoors alone with difficulty, and 95 did go outdoors alone. Those reporting not to have walked outdoors (*n* = 81) during the past 14 days were classified as “not outdoors,” and those who reported that they had walked outdoors either alone, alone with difficulty, or with assistance (*n* = 164) were classified as “outdoors.” We also divided the sample into “not outdoors alone,” those reporting that they had not been walking outdoors or had been walking outdoors with assistance (*n* = 114), and “outdoors alone” if reporting walking outdoors alone or alone with difficulty (*n* = 131).

### 2.3. Statistical Analysis

Data were checked for normality by visual inspection of Q-Q plots and the Kolmogorov-Smirnov test. Results are reported as means and standard deviations (SD). The association between physical behaviour and outdoor mobility was assessed by Spearman's correlation coefficients. Differences in physical behaviour between groups were analysed using independent samples* t*-tests and Mann-Whitney* U* tests, and *p* values <0.05 were considered statistically significant.

The Receiver Operating Characteristic (ROC) curves for all six measures of physical behaviour were plotted to discriminate both “outdoors” from “not outdoors” and “outdoors alone” from “not outdoors alone.” Sensitivity was defined as the probability of correctly classifying “outdoors” and “outdoors alone” and specificity was defined as the probability of correctly classifying “not outdoors” and “not outdoors alone.” The area under the ROC curves (AUC) is the product of sensitivity and specificity, where 1.0 represents perfect classification of the outdoor mobility question, and values of ≥0.90 are considered excellent, 0.80–0.89 good, 0.70–0.79 fair, and <0.70 poor [19]. The AUC was used to evaluate overall performance of each physical behaviour outcome measure. For each measure a cut-off point with sensitivity as close to 80% as possible was selected and used as the optimal cut-off point for that measure. All analyses were performed using IBM SPPS statistics 19.0.

## 3. Results

The 245 participants had a mean age of 83.1 years (SD 5.9) and 76% were women. Femoral neck fractures occurred in 153/245 (62.4%). Their prefracture BADL score was 18.6 (SD 2.2) and NADL score was 45.7 (SD 16.9). Detailed information about participants' physical behaviour and characteristics 12 months after surgery are presented in Table 1.

**Figure 1:**
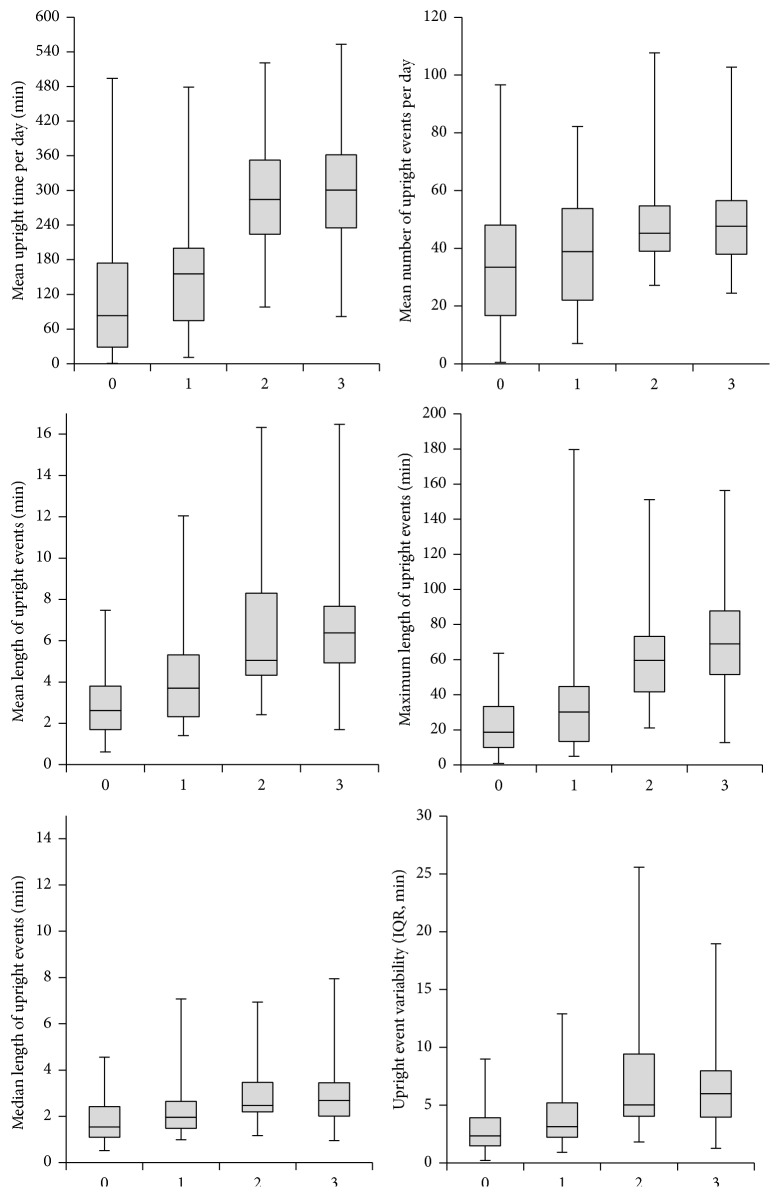
Physical behaviour in the four groups based on the single question from NADL. The boxplots show the lower quartiles, median, and upper quartiles, and the whiskers show the minimum and maximum values, for the six outcomes of physical behaviour. ^*∗*^0: not walking outdoors; 1: did go walking outdoors with personal assistance; 2: did go walking outdoors alone with difficulty; 3: did go walking outdoors alone. Those reporting 0 were classified as “not outdoors” versus 1, 2, and 3 as “outdoors”; those reporting 0 and 1 were classified as “not outdoors alone” versus 2 and 3 as “outdoors alone.”

The results for the six measures of physical behaviour for the four subgroups based on level of self-reported outdoor mobility are shown in Figure 1. Scores on the outdoor mobility scale and outcome measures of physical behaviour showed positive correlation: mean upright time (*r* = 0.61, *p* < 0.001), number of upright events (*r* = 0.36, *p* < 0.001), mean length of upright events (*r* = 0.58, *p* < 0.001), median length of upright events (*r* = 0.37, *p* < 0.001), maximum length of upright events (*r* = 0.67, *p* < 0.001), and upright event variability (*r* = 0.52, *p* < 0.001).

**Figure 2:**
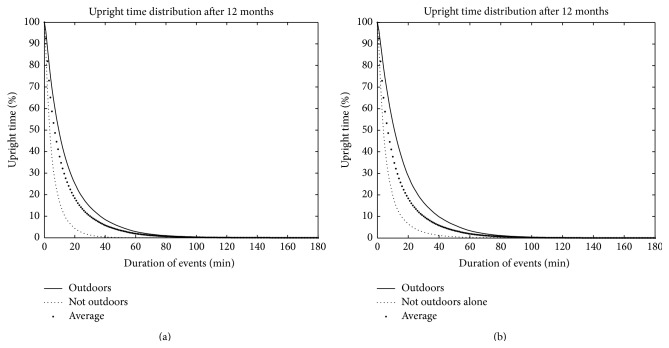
Plot of distributions of time according to lengths of upright event, where outdoors versus not outdoors (a) and outdoors alone versus not outdoors alone (b) are shown. The distribution for the average participant is shown in both figures (black dots in the middle). The percentage of upright time (*y*-axis) by lengths of upright events from shortest to longest (*x*-axis) shows that more of the total upright time was spent in longer upright events for the outdoors/outdoors alone versus not outdoors/not outdoors alone.

Furthermore, the six measures of physical behaviour were all significantly different between groups regardless of the classification used, “outdoors” versus “not outdoors” and “outdoors alone” versus “not outdoors alone” (*p* < 0.001); for details see Table 2(a).  Figure 2 shows the distribution of the length of upright events in the total sample and in the “outdoors” versus “not outdoors” and the “outdoors alone” versus “not outdoors alone.”

Cut-off points of outdoors mobility for mean upright time per day, mean number of upright events per day, mean length of upright events, median length of upright events, maximum length of upright events, and upright event variability are reported in Table 2(b). Maximum length of upright events provided the best cut-off points for both “outdoors” and “outdoors alone” (AUC = 0.87 and 0.89, resp.). If the maximum length of upright events was above 31 minutes it was more likely that subjects reported that they walked outdoors, with a sensitivity of 80.5% and specificity of 70.4%. For those with independence in outdoor walking, levels above 41 minutes for maximum length of upright events showed a sensitivity of 80.2% and specificity of 83.3%. Good classification accuracy was also shown for mean upright time per day, mean length of upright events, and upright event variability, with sensitivity of >0.80 and specificity of >0.60 for correct classification of outdoor mobility. Number of upright events per day and median length of upright events showed fair classification accuracy (AUC > 0.70, 95% CI from 0.62).

## 4. Discussion

This study investigated the relation between self-reported outdoor mobility and monitored physical behaviour in older people one year after hip fracture in order to see how well physical activity monitoring can be used to estimate outdoor walking.

Hip fractures in older persons are associated with low levels of daily physical behaviour and a loss of outdoor mobility. Participants in this study had a mean upright time of 3 hours and 36 minutes, an average of 44 transitions to upright per day, and only half of them reporting that they had been outdoors alone (53%) one year following the hip fracture. On average participants' longest upright event was almost 50 minutes. However, the mean length of upright events was just below five minutes with a variability of almost 5 minutes. For this relatively inactive sample the ability to walk outdoors would represent an important function in their daily life of great importance for independence in activities of daily living.

To our knowledge, this study is the first study to evaluate if we can derive context from activity monitoring data. In this study, all the six chosen outcome measures of physical behaviour could discriminate if a participant reported to have walked outdoors and cut-off points for all measures were therefore determined. Maximum length of upright events provided the best cut-off for outdoor mobility, with a specificity of 80-81% and a sensitivity of 70–80%. The high classification accuracy could be because a person's maximum length of an upright event might be more closely related to outdoor walking episodes as compared to the other measures of physical behaviour included in this study.

Walking time was not included as a separate outcome in this study, because the monitor's ability to detect steps for older persons walking at very slow gait speeds has been shown to be inaccurate [18]. We therefore used upright time, including both standing and walking. Upright time is a commonly used measure of physical behaviour reported in studies of older persons [3, 20], and results from this study showed that this outcome was a good discriminant of self-reported outdoor mobility in this population. Walking related outcomes from activity monitoring could possibly be even more relevant measures, especially when outdoor walking is of interest.

This study has several limitations. First, we did not consider use of walking aids in the analyses. The single question from NADL only included three answers for those walking outdoors, distinguishing walking alone from walking with difficulty from walking with assistance [10]. In groups 2 and 3 (Figure 1), it would have been interesting to identify those using walking aids, knowing that walking aids could be marker for impairment in older persons who report no difficulty when walking [21]. We also only used six measures of physical behaviour all related to upright periods and therefore consider this paper as an initial first step. Furthermore, we assessed outdoor walking as self-report, which may be affected with recall bias when assessed over a period of 14 days in this old and relatively frail population.

Further work should look into the different measures of physical behaviour and how levels and patterns of these measures are associated with physical function. This will allow clinicians to quantify patterns of physical behaviour important for prevention of functional decline in older populations.

This study confirmed that self-reported outdoor mobility and monitored physical behaviour are related. This study is however the first step in demonstrating that activity monitoring can be used to indicate if a person walks outdoors or not. Based on the data we cannot detect the exact periods of outdoor activity and cannot thus quantify the amount and pattern of the outdoor activity.

Activity monitoring provides information that is valuable because it is different from what can be obtained using assessment of physical function by self-report. Future studies should explore measures of physical behaviour more in detail, especially related to amount or level of activity needed to maintain outdoor mobility in older age.

## 5. Conclusion

Objective accelerometer-measured physical behaviour can provide important information related to outdoor mobility. The suggested cut-off points for the six physical behaviour measures in this study can be used to distinguish persons who usually walk outdoors from persons who do not walk outdoors, particularly for those who walk outdoors independently from those who do not walk outdoors independently. If a person spends long periods above 41 minutes upright, he or she is likely to be undertaking independent outdoor mobility (specificity of 80% and sensitivity of 83%). Furthermore, the six cut-off points can be used as reference values, providing quantitative information about physical behaviour related to outdoor mobility that may be useful for clinicians aiming at maintaining outdoor mobility in older people.

## Figures and Tables

**Table 1:** Sample characteristics 12 months after hip fracture surgery.

	Number of subjects	Value	Spread
	*N*	Mean (SD)	(Range)
Physical behaviour:			
Mean upright time per day (min)	245	215.5 (133.6)	(0.3–553.2)
Mean number of upright events per day	245	43.8 (19.9)	(0.5–107.7)
Mean length of upright events (min)	245	4.8 (2.8)	(0.6–16.5)
Median length of upright events (min)	245	2.4 (1.3)	(0.52–7.95)
Maximum length of upright events (min)	245	48.9 (35.2)	(0.84–179.6)
Upright event variability (IQR, min)	245	4.8 (3.4)	(0.2–25.6)
Mobility:			
TUG 12 months (sec)	225	22.3 (15.8)	(7.8–126.9)
SPPB (0–12)	241	5.3 (3.3)	(0–12)
Gait Speed (m/s)	231	0.63 (0.25)	(0.07–1.42)
ADL function:			
BI (0–20)	245	16.9 (3.8)	(4–20)
NADL (0–66)	245	34.4 (19.3)	(1–66)
Cognitive function, MMSE (0–30)	243	24.0 (5.0)	(5–30)
Depression, GDS (0–15)	235	4.1 (3.2)	(0–13)
Grip Strength (kg)	234	21.6 (8.0)	(4–54)
Fear of Falling, FES-I (7–28)	231	11.1 (4.1)	(7–28)

^*∗*^Mean upright time per day (min): total minutes of all upright events/number of recording days; mean number of upright events per day: average number of upright events per day; mean length of upright events: average length in minutes based on all upright events during the recording period; maximum length of upright events: maximum length in minutes for the longest upright event during the recording period; median length of upright events: median length in minutes based on all upright events during the recording period; upright event variability: the interquartile range (IQR) of upright events lengths in minutes during the recording period; TUG: Timed Up-and-Go; SPPB: Short Physical Performance Battery; Gait Speed: based on the 4-meter gait test from SPPB; BI: Barthel Index; NADL: Nottingham Extended Activities of Daily Living scale; MMSE: Mini Mental State Examination; GDS: geriatric depression scale; Grip Strength: measured by the JAMAR dynamometer in kg; FES-I: the 7-item Fall Efficacy Scale International FES-I.

**(a):** 

	“Not outdoors” versus “outdoors”					“Not outdoors alone” versus “outdoors alone”				
	Group 0, Figure 1		Groups 1, 2, and 3, Figure 1		Independent	Groups 0 and 1, Figure 1		Groups 2 and 3, Figure 1		Independent
	(*n* = 81)		(*n* = 164)		*t*-test	(*n* = 114)		(*n* = 131)		*t*-test
	Mean	(SD)	Mean	(SD)	*p* =	Mean	(SD)	Mean	(SD)	*p* =
Mean upright time per day (min)	114.8	(100.3)	265.2	(119.4)	<0.001	127.9	(104.1)	291.7	(107.2)	<0.001
Mean number of upright events per day	35.0	(21.9)	48.2	(17.3)	<0.001	36.2	(21.3)	50.4	(15.9)	<0.001
Mean length of upright events (min)	2.9	(1.5)	5.8	(2.8)	<0.001	3.3	(1.9)	6.2	(2.7)	<0.001
Maximum length of upright events (min)	21.7	(14.7)	62.3	(34.7)	<0.001	26.3	(23.7)	68.5	(31.8)	<0.001
Median length of upright events (min)	1.8	(0.9)	2.7	(1.3)	<0.001	2.0	(1.1)	2.8	(1.3)	<0.001
Upright event variability (min)	2.8	(1.7)	5.8	(3.5)	<0.001	3.2	(2.2)	6.3	(3.5)	<0.001

^*∗*^The NADL question used was if the participants had been walking outdoors the past 14 days. “Not outdoors”: participants who had not walked outdoors; “outdoors”: participants who had walked outdoors either alone, alone with difficulty, or with assistance; “not outdoors alone”: participants who had not walked outdoors or walked outdoors with assistance; “outdoors alone”: participants who reported walking outdoors alone or alone with difficulty. Mean upright time per day (min): total minutes of all upright events/number of recording days; mean number of upright events per day: average number of upright events per day; mean length of upright events: average length in minutes based on all upright events during the recording period; maximum length of upright events: maximum length in minutes for the longest upright event during the recording period; median length of upright events: median length in minutes based on all upright events during the recording period; upright event variability: the interquartile range (IQR) of upright events lengths in minutes during the recording period.

**(b):** 

Measure of PB	Outdoor mobility					Independent outdoor mobility				
	AUC	95% CI	SENS	SPEC	CP	AUC	95% CI	SENS	SPEC	CP
Mean upright time per day	0.836	0.78–0.89	0.805	0.716	157.7	0.865	0.82–0.91	0.802	0.754	198.9
Mean number of upright events per day	0.697	0.62–0.77	0.805	0.531	34.8	0.707	0.64–0.77	0.802	0.526	36.8
Mean length of upright events	0.834	0.78–0.89	0.811	0.679	3.4	0.838	0.79–0.89	0.802	0.719	4.0
Maximum length of upright events	0.871	0.83–0.91	0.805	0.704	30.8	0.891	0.85–0.93	0.802	0.833	41.2
Median length of upright events	0.732	0.66–0.80	0.805	0.519	1.7	0.719	0.66–0.78	0.802	0.535	1.8
Upright event variability	0.804	0.75–0.86	0.805	0.605	3.2	0.810	0.76–0.86	0.802	0.640	3.5

^*∗*^AUC: area under receiver operating characteristic curve; CP: cut-off point for predictors above which subject is more likely to report that they are outdoor walking; *n*: sample; SENS: sensitivity; SPEC: specificity; PB: physical behaviour; mean upright time per day: total minutes of all upright events/number of recording days; mean number of upright events per day: average number of upright events per day; mean length of upright events: average length in minutes based on all upright events during the recording period; maximum length of upright events: maximum length in minutes for the longest upright event during the recording period; median length of upright events: median length in minutes based on all upright events during the recording period; upright event variability: the interquartile range (IQR) of upright events lengths in minutes during the recording period.
